# Spatial heterogeneity of climate explains plant richness distribution at the regional scale in India

**DOI:** 10.1371/journal.pone.0218322

**Published:** 2019-06-20

**Authors:** Poonam Tripathi, Mukunda Dev Behera, Partha Sarathi Roy

**Affiliations:** 1 Centre for Oceans, Rivers, Atmosphere and Land Sciences (CORAL), Indian Institute of Technology Kharagpur, West Bengal, India; 2 NASI Senior Scientist Platinum Jubilee Fellow, System Analysis for Climate Smart Agriculture, Innovation Systems for the Dry lands, ICRISAT, Pathancheru, Hyderabad, India; UNAM, MEXICO

## Abstract

**Introduction:**

Knowledge of species richness patterns and their relation with climate is required to develop various forest management actions including habitat management, biodiversity and risk assessment, restoration and ecosystem modelling. In practice, the pattern of the data might not be spatially constant and cannot be well addressed by ordinary least square (OLS) regression. This study uses GWR to deal with spatial non-stationarity and to identify the spatial correlation between the plant richness distribution and the climate variables (i.e., the temperature and precipitation) in a 1° grid in different biogeographic zones of India.

**Methodology:**

We utilized the species richness data collected using 0.04 ha nested quadrats in an Indian study. The data from this national study, titled ‘Biodiversity Characterization at Landscape Level’, were aggregated at the 1° grid level and adjudged for sampling sufficiency. The performances of OLS and GWR models were compared in terms of the coefficient of determination (R^2^) and the corrected Akaike Information Criterion (AICc).

**Results and discussion:**

A comparative study of the R^2^ and AICc values of the models showed that all the GWR models performed better compared with the analogous OLS models. The climate variables were found to significantly influence the distribution of plant richness in India. The minimum precipitation (Pmin) consistently dominated individually (R^2^ = 0.69; AICc = 2608) and in combinations. Among the shared models, the one with a combination of Pmin and Tmin had the best model fits (R^2^ = 0.72 and AICc = 2619), and variation partitioning revealed that the influence of these parameters on the species richness distribution was dominant in the arid and the semi-arid zones and in the Deccan peninsula zone.

**Conclusion:**

The shift in climate variables and their power to explain the species richness of biogeographic zones suggests that the climate–diversity relationships of plants species vary spatially. In particular, the dominant influence of Tmin and Pmin could be closely linked to the climate tolerance hypothesis (CTH). We found that the climate variables had a significant influence in defining species richness patterns in India; however, various other environmental and non-environmental (edaphic, topographic and anthropogenic) variables need to be integrated in the models to understand climate–species richness relationships better at a finer scale.

## Introduction

Knowledge of plant richness patterns under various environmental conditions is important in dealing with biodiversity conservation and management actions. The spatial distribution of species is associated with variations in latitude, elevation, climate and area [1–4]. Among these, the climate variables, i.e. precipitation (water) and temperature (energy), have emerged as the key influencing factors [5–7]. Water and energy are essential for plant physiological processes as they directly influence photosynthesis, respiration, plant growth and productivity [8]. However, the influences of the two factors (water and energy) may not be equally important globally, and their relative importance shifts along a latitude [8–10]. Many plausible hypotheses have been postulated on the basis of these findings and observations. The energy hypothesis suggests that the species richness of a region is a function of the total energy available and, therefore, provides a positive relationship between the species richness and energy variables such as temperature [11–13]. Another important insight into the water–energy dynamics was provided by O’Brien [14]. This insight suggests that the broad-scale patterns of species richness derive from the interaction of the available energy and water. It is predicted from the water–energy dynamics that more species occupy regions where more water and energy are available and that the strength of this relationship might vary with spatial scale [15, 16]. The climate tolerance hypothesis (CTH) suggests that ‘species richness is the highest at warm and/or humid environment[s] because a wider range of functional strategies can persist under similar conditions’ [6]. Several species cannot survive in extremely cold or hot environments [17]. The environmental stress hypothesis suggests that the species pool decreases with increasing climate harshness [18]. Climatic harshness is often defined by extreme climatic variability, e.g. low temperatures and low water availability [6]. On the basis of the aforementioned hypotheses, it may be inferred that the spatial heterogeneity of climatic factors may significantly influence the species distribution pattern at both the regional and global scales. Therefore, the distribution of plant richness is not smooth, and different drivers may apply at different latitudes or in different biogeographic regions [8, 13].

India consists of ten major biogeographic zones, which are characterized by peculiar climates, for example, the very dry desert zone in western India and the wet Western Ghats zone in south-western India. The varying patterns of species in these zones might be attributed to non-uniform climatic variables. The climate has historically been varying, leading to spatial heterogeneity [19–21]. Thus we may expect geographical shifts in the explanatory power of climate predictors, from south to north and from east to west. However, an implicit assumption of spatial non-heterogeneity has been found in statistical analysis, and consequently, predictions and hypothesis testing based on global models (models whose parameters, such as the slope and intercept, are used universally) are precluded [22, 23]. Global models such as the ordinary least square (OLS) method ignore spatial autocorrelation and thus assume spatial non-heterogeneity and constant variance across space. This results in biased estimates of parameters [24], misleading significance tests and sub-optimal predictions [25]. Many statistical models have been developed as alternatives that accommodate spatial heterogeneity without compromising universal model parameters. Geographically weighted regression (GWR) modelling has gained importance as it explicitly incorporates the issue of spatial heterogeneity and calculates regression parameters for each local unit (e.g., grid or cell) by allowing the depiction of an individual predictor’s performance across space [25]. This helps reveal the spatial variation of the relationship between variables, which would otherwise be ignored.

In the present study, our goal was to assess the spatial relationship between the climate variables and the vascular plant richness of India in different biogeographic zones. We studied the spatial autocorrelation of the species richness with two climate variables (i.e. precipitation and temperature) using non-stationarity by comparing a global model with a local model (i.e., OLS and GWR). Precipitation and temperature are two of the variables most commonly considered in studies to address how the climate shapes the plant community distribution and diversity at the global scale [26, 27]. We utilised the Indian national-level plant species database generated through the project ‘Biodiversity Characterization at Landscape Level’ [28]. The quadrat-level (0.04 ha) richness was observed to range from 1 to 59, and we aggregated this quadrat-level richness at two grid levels to assess the sampling completeness [29]. In the present study, we used only the sufficiently sampled grids (219 out of 301) at 70% of the sampling completeness threshold given by Tripathi et al. [29], who fitted the asymptotic clench model to assess sampling completeness. We chose temperature and precipitation and analysed them across the various biogeographic zones of India.

## Materials and methods

### Study area

**Fig 1:**
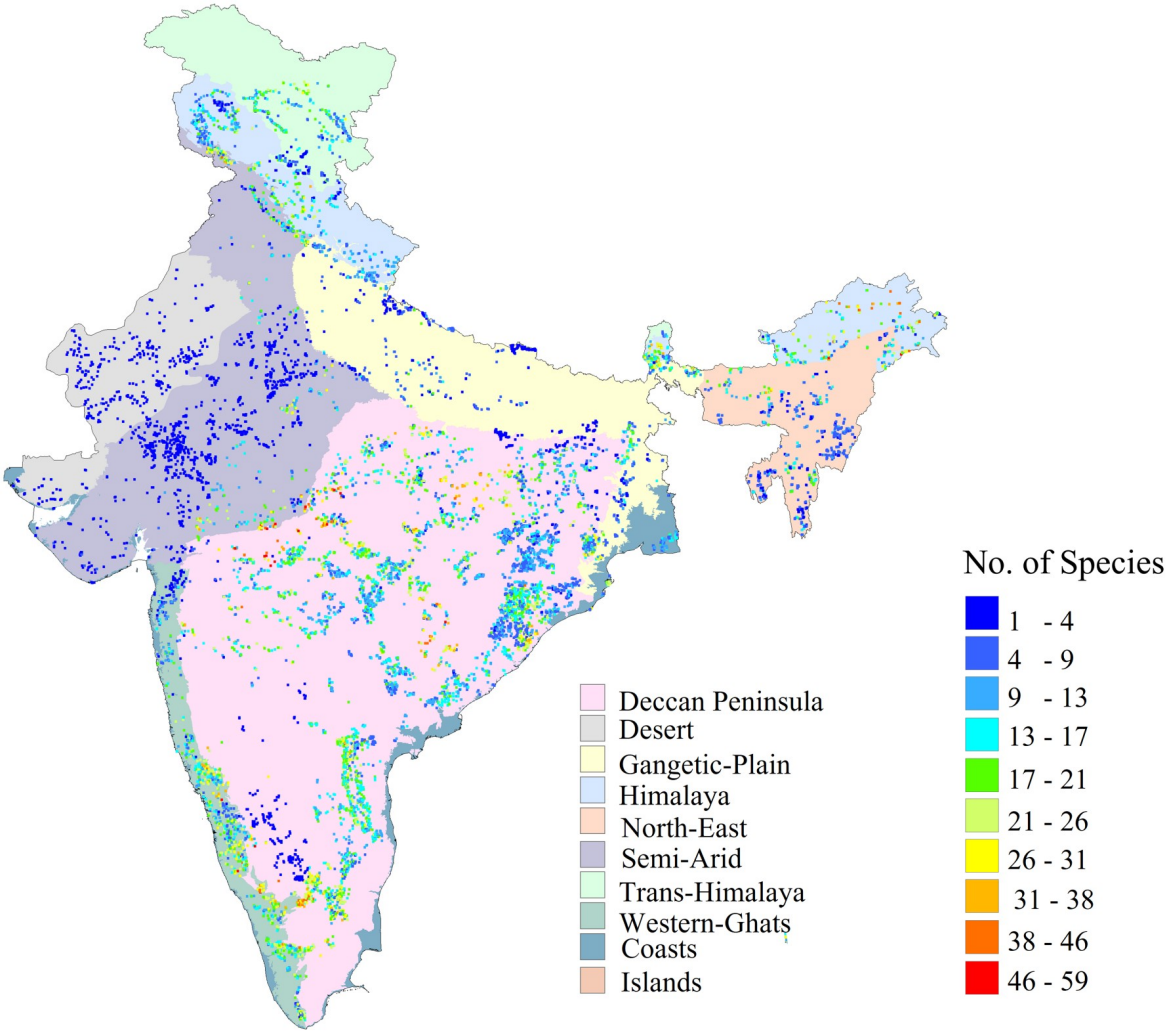
Species distribution in India against the backdrop of major biogeographic zones as per Rodgers and Panwar (1988).

The study area, the Indian mainland, with a total geographical area of nearly 329 million hectares, lies to the north of the equator, between latitudes 6° 44′ and 35° 30′ N and longitudes 68° 7′ and 97° 25′ E (Fig 1). The Indian climate varies from temperate in the north to monsoonal in the south, and accordingly, four major monsoonal periods are recognisable: (i) the essentially warm and humid south-west summer monsoon (June–August), (ii) cold and dry north-east winter monsoon (December–February), (iii) spring (March–May) and (iv) autumn (September–November). Most of the annual rainfall (80%) is received during the south-west summer monsoon. The great spatial variability in monsoonal activity has accounted for the diverse biogeographic zones, with a wide range of vegetation types and distinctive ecological regimes, biomes, communities and species (Fig 1 [30]). Major biodiversity hotspots are located in these zones, e.g. the Western Ghats, the Himalaya and the Trans-Himalaya zones [31]. The Himalaya and the Trans-Himalaya zones are at higher elevations, ranging from 500 m to nearly 8000 m. These zones are highly fragile due to extreme climatic and topographic variations [30]. Dry alpine pasture is the major vegetation class in the Trans-Himalaya region. The Himalaya zone consists of moist temperate forest, temperate conifer, pine and evergreen forests and scrub land. As a result of the high rainfall and rugged terrain of the region, there is less anthropogenic pressure. The arid and the semi-arid zones are characterized by dry climatic conditions owing to low rainfall (400–1000 mm), and the major vegetation types are dry deciduous and thorn forests. The Deccan peninsula zone is the most extensive zone. The vegetation is of the deciduous type, and the biological species richness is greater in the hill ranges of the Eastern Ghats. The Western Ghats zone comprises two major vegetation divisions: the western portion is characterised by very high rainfall and is covered with evergreen forests, while the eastern belt, in the rain shadow, consists largely of deciduous forests [32]. The Gangetic plains zone is the most fertile region of the world, but it is highly populated. As a result, the forests in the region have been cleared. There is a clear west–east moisture gradient, with the rainfall ranging from 500 mm to 5000 mm, in the region. The North-eastern zone is one of the regions with the richest biodiversity, and it is also characterised by a high level of disturbance. The forests are privately owned by people, and therefore *jhum* cultivation (slash-and-burn agriculture) is prominent in this zone [33]. In spite of this diversity, no study has been conducted on the spatial relationship between the plant richness and climate at the national scale in India, perhaps due to a lack of systematic data on the plant richness distribution [34, 35].

### Species richness data

We used the species richness data collected through the project ‘Indian National-Level Biodiversity Characterization at Landscape Level’ [28]. This project was carried out over a 12 year span from 1998 to 2010. The vegetation types were classified and different attributes of species richness were developed into a spatial database in this project [36]. A total of 15,565 nested quadrats of area 0.04 ha (20×20 m^2^) each were laid in 100 vegetation types across the length and breadth of the country, 15,527 quadrats being in the Indian mainland (excluding the Andaman & Nicobar and Lakshadweep islands). The sites were selected on the basis of stratified random sampling (Fig 1 [28]). To sample shrub species, two plots of size 5 ×5 m^2^ each were laid at opposite corners of tree plots, and for herb species, five plots of size 1×1 m^2^ each were laid at the four corners and centres of tree plots (S1a Fig). The quadrat-level data displayed in S1b Fig were aggregated at 1° grids to assess the sampling sufficiency according to the method of Tripathi et al. [29]. We analysed the species richness distribution patterns of various biogeographic zones. We did not consider the coastal region because of its narrow spatial extent in the 1° grid (Fig 1). Nearly 80% of the grids were sufficiently sampled (at the 70% sufficiency threshold) [29] and were included in the statistical analysis of this study.

### Spatial correlation of species richness with climate

#### Climate data

Six environmental (predictor) variables, namely the mean annual temperature (MAT), mean temperature of the coldest month (Tmin), mean temperature of the warmest month (Tmax), mean annual precipitation (MAP), mean precipitation of the driest month (Pmin) and mean precipitation of the wettest month (Pmax), were used in this study. The values of these variables were calculated for the data sets of the past 100 years. The data were obtained from climatic products of the Climatic Research Unit (CRU) at a resolution of 0.5° × 0.5°. New et al. [37] derived these data from monthly terrestrial surface climate variables for the period from 1901 to 1998 (CRU TS 1.0) and updated them to 2000 (CRU TS 1.1). Mitchell et al. [38] revised the grids and updated them to 2000 (CRU TS 2.0). The present species richness of a region is potentially the result of the adaptation of the species to the past climate and their inheritance of climatic niches from their ancestors. Therefore, the past climate can provide useful insights of relevance to the species distribution pattern with respect to the climate pattern [39].

Here, MAP is the averaged monthly precipitation over 100 years, and MAT is the monthly averaged temperature of 100 years. The Tmin and Tmax values were the averaged minimum and maximum values of the coldest and warmest months, respectively, of the past 100 years. Pmin and Pmax were the averaged monthly rainfall values of the driest month and the wettest month, respectively. All the climatic variables were resampled to 1° × 1° to match the species richness data grids. The temperature and precipitation data were also used to derive climate anomalies to see the long-term climatology and its methodology is presented in S1i Appendix.

#### Global and local models

We estimated a global model for each predictor variable using OLS to serve as a baseline for subsequent local analyses. The assumption of the global regression method is that the relationship under study is stationary and therefore the estimated parameters remain constant in space. This regression model relies on determining the dependent variable (y) by producing a minimum sum of squares with regard to the independent variable, x. OLS works on a set of assumptions such as normality, homogeneity and independence of residuals and can be described by the equation

y=β0+β1×x+ε (1)

where β0 is the intercept of the line on the y axis (where x = 0), β1 represents the slope coefficient of the independent variable, x, and ε is the deviation of the point from the regression line. Regression fitting involves estimation of a and β for the best fit by minimising the total error, Σε^2^.

A GWR (an extended version of OLS) analysis was performed for each set of variables to account for non-stationarity in the species richness–climate relationships. GWR calibrates multiple regressions and fits them at each sample point. This approach allows all regression statistics such as partial slopes and the coefficient of determination (R^2^) to be calculated at each focal cell. The fitting is based on a spatial proximity approach. Here, proximity is defined as the set of locations within a given bandwidth (b) of pre-set radius and shape [25]. The contribution of each observation to the GWR analysis at a specific location is dependent on its geographical distance from that location, with distant observations having less impact compared with nearby ones. We implemented this geographical weighting through a spatial kernel function, the bandwidth of which determines the scale of analysis. The regression equation is:

$$\text{Yi}=\beta_{0}\left({\text{u}}_{\text{i}}\;{\text{v}}_{\text{i}}\right)+\sum_{\text{j}=1}^{\text{k}}\left({\text{x}}_{\text{ij}}\beta_{\text{j}}\left({\text{u}}_{\text{i}},{\text{v}}_{\text{i}}\right)+\varepsilon_{\text{i}}\right)$$ (2)

where j = 1, k are explanatory variables, ε_i_ is a random error term, and the location of each observation is defined by the coordinates (u_i_, v_i_). β_0_–β_k_ are the parameters of the model, with β_j_(u_i_,v_i_) being a realization of the continuous function β_j_(u_i_,v_i_) at location i.

**Fig 2:**
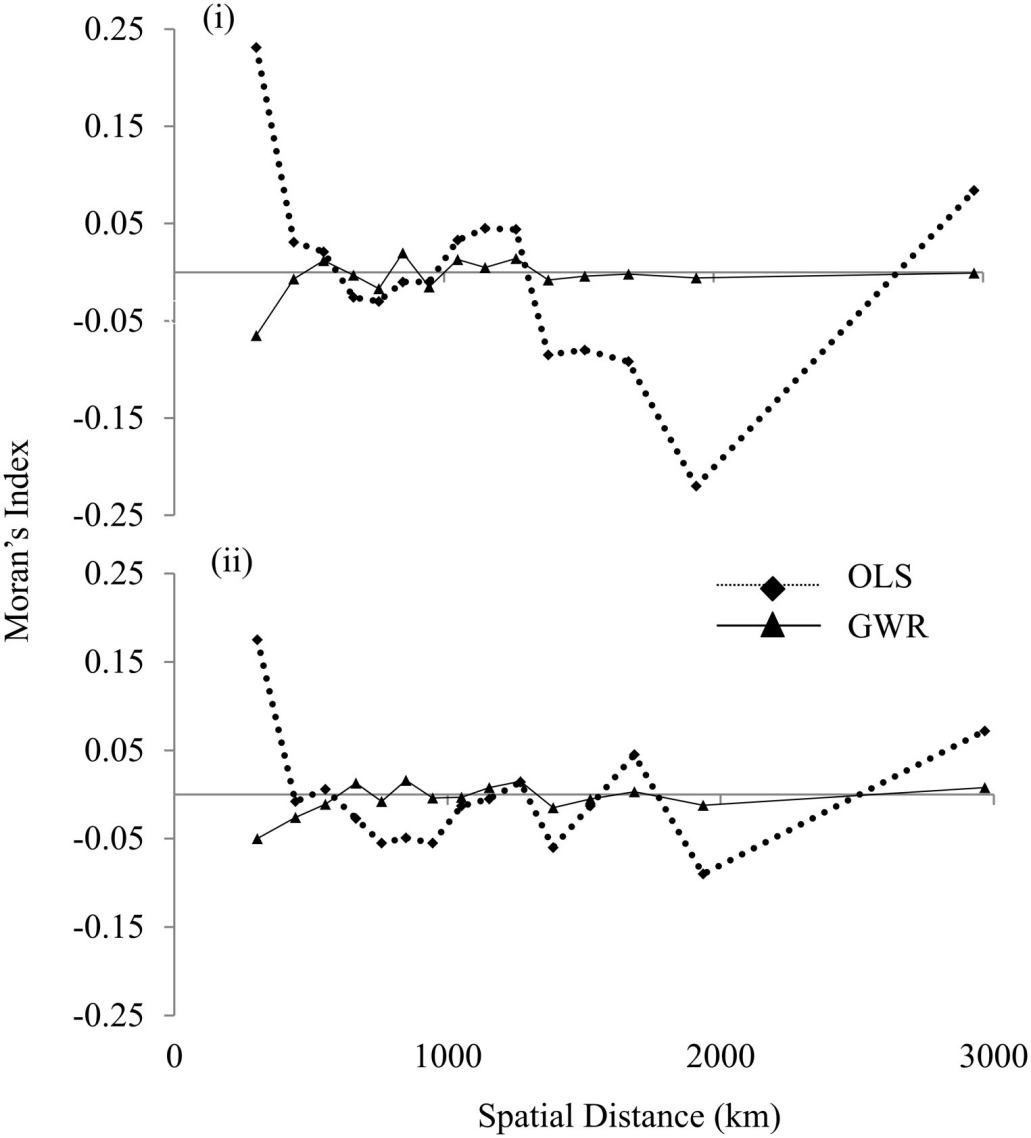
Spatial correlograms for the residuals of (i) Tmin and Pmin and (ii) all six variables using OLS and GWR models.

**Fig 3:**
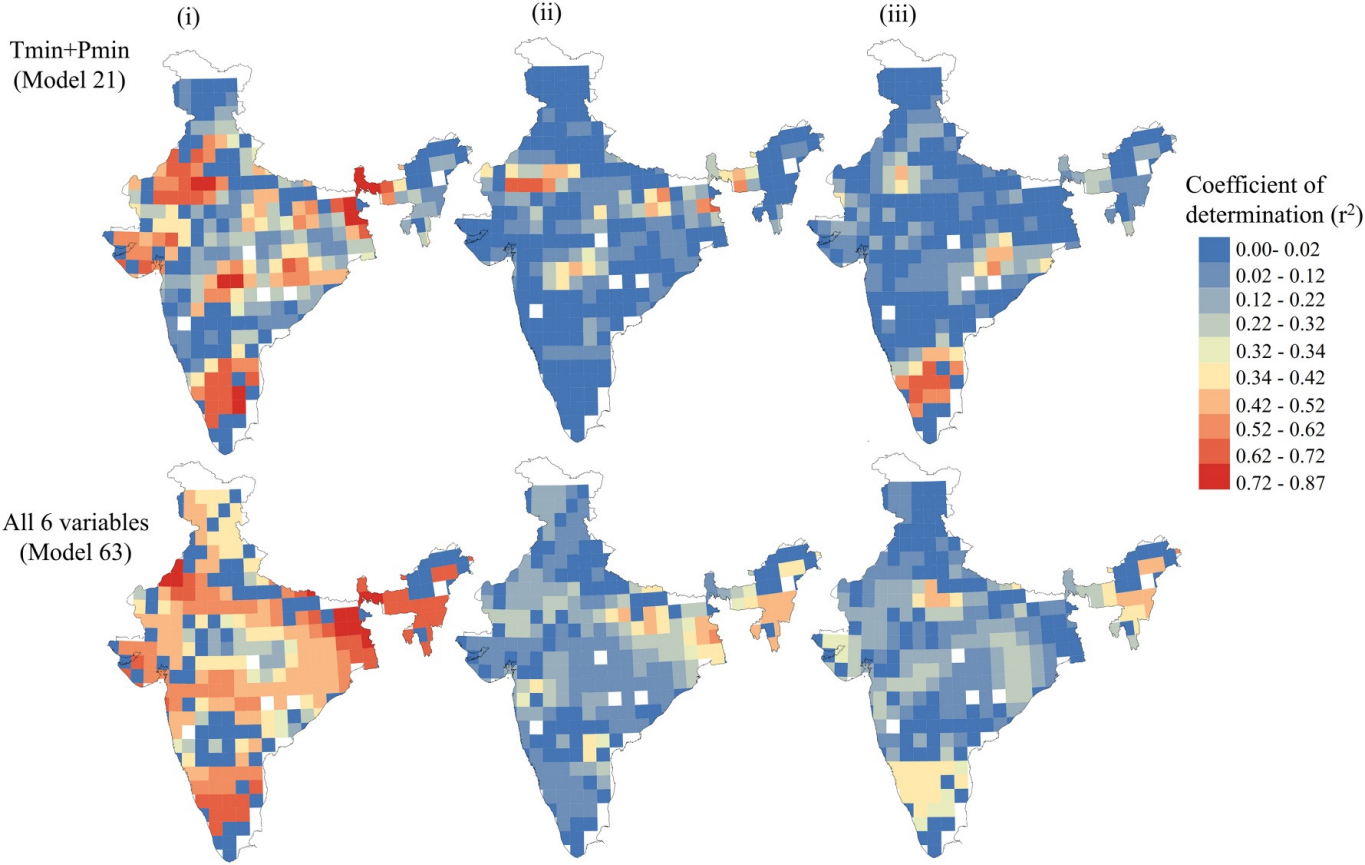
Variation partitioning showing the correlation of plant species richness with (i) temperature and precipitation (i.e., energy and water), (ii) temperature only and (iii) precipitation only for two different combinations of models.

As pointed out by Fotheringham et al. [25], a fixed kernel function might mask the subtle spatial non-stationarity in dense data clusters and might exaggerate the degree of non-stationarity. Therefore, an adaptive bi-square spatial kernel weighting function was used to avoid large local estimation variances in areas where the data are sparse. We analysed four neighbours at 5% increments, from 5% to 20%, to capture the spatial variation, where the 5% of neighbor represents the least number of neighboring grid cells and vice versa. The corrected Akaike information criterion (AICc) was used to compare the performance of OLS regression with GWR-based outputs. As a general rule, a lower AICc value indicates a better fit. Thus, the best model has the lowest AICc value [25]. All the possible combinations of the predictor variables (represented as models) were designed to predict the plant richness (response variable). However, only the best models, with combinations of predictor variables depicting more close results to observations, have been shown in Figs 2 and 3 and Table 1. The details are provided in S1 Table. Moran’s I was used as a measure of the spatial autocorrelation of the regression residuals. Moran’s index has an expected value of near-zero under the null hypothesis of no spatial autocorrelation. However, positive and negative values show strong positive and negative autocorrelation, respectively. The values of Moran’s I autocorrelation of the residuals from each regression model were computed and compared to decide if one model was better than the other (Fig 2). Strong spatial collinearity was observed in the variables MAP and Pmax (S2i Fig), the influence of which was dealt with by using variation partitioning. For the purpose, we built shared models by combining different variables in a single model (S1 Table). Further, we computed local R^2^ values separately for each variable and for the shared model, where the R^2^ values represent the local variation in species richness.

**Table 1:** Descriptive statistics of temperature, precipitation and combinations of their variables shown as their coefficient as derived from OLS and GWR models.

OLS					GWR					
Model	Parameter	Estimate	S.E.	2 S.E.	Minimum	25% quartile	Median	75% quartile	Maximum	IQR
M1	Intercept	278.18	30.67	61.35	-2206.22	163.85	368.82	1039.82	3309.50	875.97
	MAT	-4.07	1.28	2.57	-123.22	-34.95	-7.27	2.03	87.95	36.98
M 2	Intercept	175.40	18.86	37.71	-504.16	13.87	121.60	248.04	915.38	234.17
	MAP	0.01	0.02	0.04	-0.57	-0.05	0.05	0.19	0.76	0.24
M 3	Intercept	325.78	41.80	83.60	-2802.42	45.39	424.31	1144.35	2908.53	1098.96
	Tmax	-4.75	1.38	2.75	-81.02	-27.97	-8.85	4.16	95.82	32.14
M 4	Intercept	213.46	18.95	37.90	-1531.58	-265.83	162.28	426.17	1805.27	691.99
	Tmin	-1.86	1.09	2.19	-91.58	-16.54	0.54	27.22	101.52	43.75
M 5	Intercept	164.85	18.01	36.02	-1189.54	-3.92	134.84	235.73	883.23	239.65
	Pmax	0.01	0.01	0.01	-0.16	-0.02	0.02	0.06	0.41	0.08
M 6	Intercept	153.15	14.63	29.26	-112.49	71.67	147.15	225.18	519.54	153.50
	Pmin	0.64	0.24	0.48	-5.39	-0.71	0.81	2.79	17.40	3.50
M 21	Intercept	161.42	31.63	63.25	-2273.69	-313.22	75.55	333.30	1782.57	646.52
	Tmin	-0.39	1.30	2.61	-82.26	-9.62	1.39	24.75	118.44	34.38
	Pmin	0.59	0.29	0.58	-7.98	-0.60	0.85	3.39	13.63	3.99
M 63	Intercept	364.89	78.94	157.87	-4057.34	-211.30	774.33	1601.07	6656.07	1812.37
	Tmax	7.88	7.36	14.72	-690.74	-65.38	-15.86	38.55	418.63	103.94
	Tmin	18.80	4.45	8.89	-890.50	-85.87	-1.81	55.41	177.90	141.28
	MAT	-30.49	10.0	20.01	-371.43	-85.13	-16.82	88.22	1302.89	173.35
	Pmax	0.02	0.02	0.04	-1.97	-0.10	0.01	0.13	3.14	0.23
	Pmin	0.52	0.35	0.70	-23.86	-2.75	0.10	2.73	15.70	5.48
	MAP	-0.11	0.06	0.12	-8.38	-0.43	0.08	0.51	6.88	0.94

**Full form of parameters**: MAT: average temperature; Tmin: minimum temperature; Tmax: maximum temperature; MAP: average precipitation; Pmin: minimum precipitation; Pmax: maximum precipitation; S.E.: standard error of mean; 2S.E.: twice of standard error of mean; IQR: Inter quartile range

Variation partitioning for each grid cell to determine the variation, which is individually explained by the precipitation and temperature and was calculated as:

$$\begin{array}{l} \text{Rp}=\text{Rc}-\text{Rt}\;\text{for}\;\text{pure}\;\text{water}\;\text{and}\\ \text{Rt}=\text{Rc}-\text{Rp}\;\text{for}\;\text{pure}\;\text{temperature}, \end{array}$$

where Rc is the local R^2^ value of the combined model and Rp and Rt represent the local R^2^ values of the precipitation and temperature, respectively. All the analyses were performed using the Spatial Analyses in Macroecology (SAM) software package [40].

## Results

### Plant richness in different biogeographic zones at 1° grids

Different ranges of species richness were observed at the 1° grid level, with the highest in the single grid of the Gangetic plain zone (623) (S1 Fig). The number of species varied between 10 and 609 in the Deccan peninsula, between 31 and 581 in the Western Ghats, between 97 and 531 in the Trans-Himalaya, between 10 and 623 in the Gangetic plains, between 14 and 160 along the coast, between 30 and 344 in the North-east, between 3 and 517 in the semi-arid zone and between 6 and 175 in the desert zone (S1 Fig & S2 Table).

### Spatial autocorrelation and climate variables

As a pre-condition of GWR, the variables should be spatially auto-correlated and can be interpreted by drawing spatial correlograms. All the variables showed positive autocorrelation over short distances and turned negative with increasing distance (S2 Fig). Positive autocorrelation was observed up to ~750 km and 800 km (P<0.001) for Tmax and Tavg, up to 1000 km and 1040 km (P<0.001) for Tmin and MAP, up to 700 km (P<0.03) for Pmin, and up to 1050 km (P<0.001) for Pmax (S2i & S2ii Fig). MAP and Pmax followed the same trend of spatial autocorrelation (S2ii Fig). The spatial variations of the variables precipitation and temperature run in opposite directions. MAT decreases from west (~26°C) to east (~12°C), whereas MAP decreases strongly from east (~170 mm) to west (12 mm; S3ii and S4ii Figs). Moving from south to north, MAT sharply decreases in the Himalaya and the Trans-Himalaya (~28°C in the south to -6°C in the north; S3ii and S4ii Figs). In general, the ranges of all the climate variables and their anomaly vary considerably in the different zones, and they are described in S1ii Appendix.

### Model evaluation and investigation of climate variables as predictors of species richness

A clear non-stationary correlation was observed between species richness and climate in India (Table 1). It is apparent here that all the OLS estimates for the parameters falling within the 25% quartile and the median range, or between the median and the 75% quartile of GWR, showed that all the local estimates are higher than the OLS values. For example, the estimates for the model M1 have an inter-quartile range of 875.97 and MAT as 36.98. These ranges are much broader than twice the standard error, i.e., 61.35 and 2.57, respectively (Table 1). This indicates that all the climate variables are better fitted by the non-stationary model GWR than using a global (OLS) regression. The local R^2^ values also favoured this spatial heterogeneity in relation to the water and energy dynamics. The GWR analyses rendered better model fits compared with OLS for all sets of variables, showing significantly smaller AICc values and the highest values of the coefficient of determination (R^2^; see S1 Table). We observed that the models performed better at 10% of neighbour for an individual variable, with the more close prediction of species richness with observed one and the lowest AICc and significant R^2^ value (S1 Table). This performance shifted to 15% and 20% of neighbours with the addition of more variables (shared model). All the variables could explain the variation of 61% to 69% individually for 10% of neighbours, and the overall correlation was improved by 2% to 3% in the shared model, wherein the spatial variation also improved by 10% to 50% (see S1 Table and Fig 3). Among the energy models (temperature variables alone), Tmin (M4) was observed to be the most supporting, with the lowest AICc (2635.9) and coefficient of determination (R^2^) (0.64) values. Among the water models (precipitation variables alone), the model considering Pmin only (M6) was observed to be more closer to reality, with the lowest AICc (2608.0) and R^2^ (0.69) values in the GWR model (S1 Table).

Among all the variables (individual and in the shared model), Pmin emerged consistently as the strongest predictor. All the other predictor variables also had comparable R^2^ values but higher AICc values (S1 Table). We selected the best model on the basis of the lowest AICc value and a good R^2^ value as there is a rule of thumb that an absolute differential AICc value of ≥3 provides clear evidence of improved model performance [25].

Among the shared model, the combination of Tmin and Pmin (M21), emerged as the best supported one, with the lowest AICc value (2619.6) and R^2^ value (0.72) at 10% of neighbour (Fig 3). The maximum R^2^ value (>0.5) was noted for the semi-arid zone, the desert zone, the southern Western Ghats and the extreme east of the Gangetic plains (Fig 3). The weakest correlation (R^2^<0.2) was observed in the border areas of the Deccan peninsula and in the Trans-Himalaya zone. Most of the grids showed a range of R^2^ values between 0.4 and 0.7 (Fig 3).

**Fig 4:**
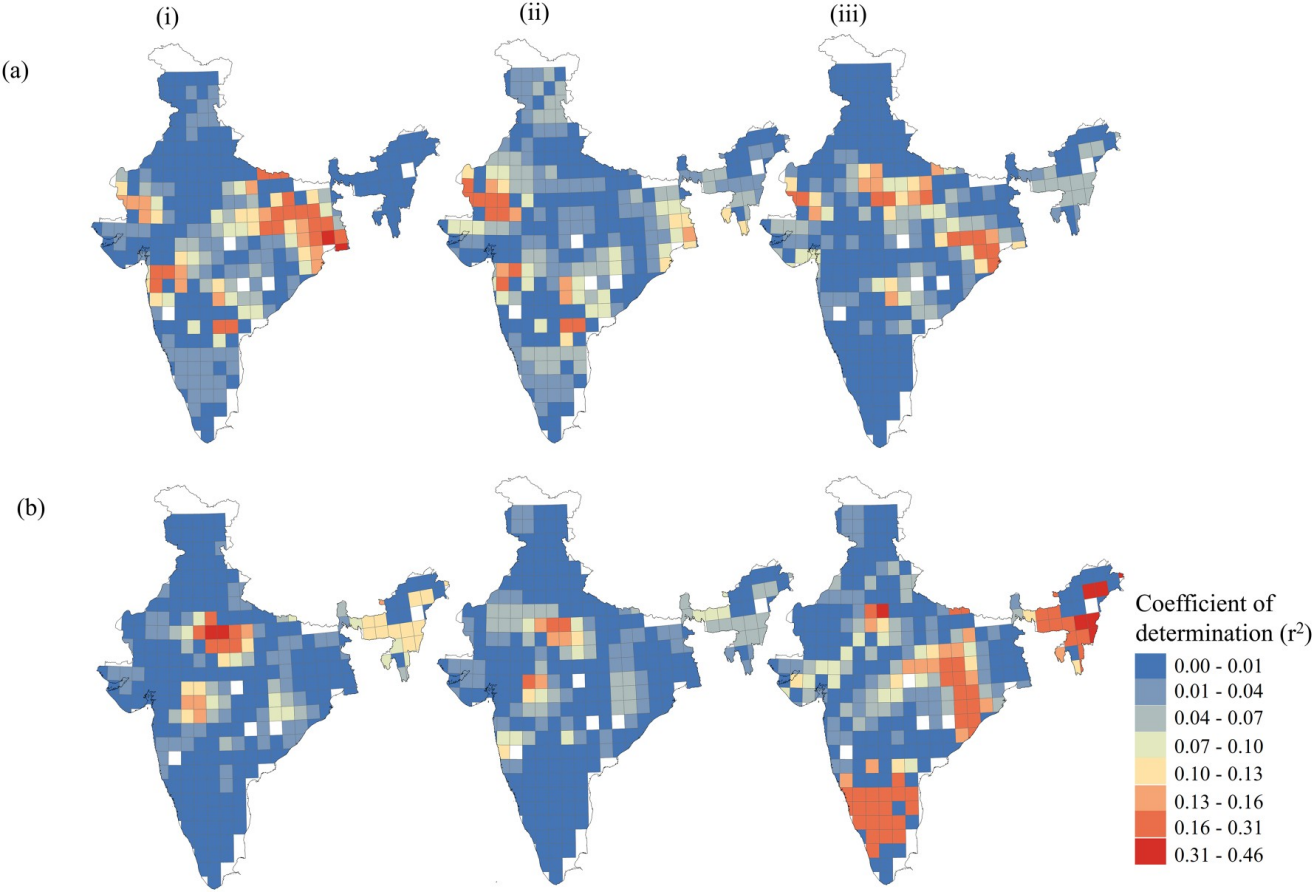
Variation partitioning showing the correlation of plant species richness with (a) temperature (energy) variables (MAT (i), Tmax (ii), Tmin (iii)) and (b) precipitation (water) variables (MAP(i), Pmax (ii), Pmin (iii)) in model M63.

A combination of all six climate variables (M63) was also analysed to determine the spatial influence of all the variables that had the minimum AICc value (2649) at the 20% neighbour and R^2^ value (0.70). Spatially, the R^2^ value ranged from 0.02 to 0.87. It is apparent from the mapping of the variation partitioning that the combination of all the climate variables (M63) represents a spatial improvement over a combination of Tmin and Pmin (M21) due to the additional contributions of the other variables. Variation partitioning revealed that the maximum variation (R^2^ variation between 0.13 and 0.46) was contributed by Pmin in the southern region of the Western Ghats, the Deccan peninsula and North-east zone (Fig 4b). In contrast, the temperature variables, i.e., MAT, Tmax and Tmin, had noteworthy influence (R^2^ value between 0.04 and 0.31) in the eastern parts of the Deccan peninsula, the desert and the semi-arid zone, the Gangetic plain and the North-east zone, respectively (Fig 4a). In general, Pmin consistently dominated in all the models, strongly supporting the notion regarding the influence of precipitation on species richness in tropical regions.

In contrast to GWR, OLS had a maximum R^2^ value of 0.14 and a minimum AICc value of 2727.5 for a combination of the variables MAT, Tmin, MAP and Pmin (M53; S1 Table). The error terms from the analysis of the residuals showed a strong autocorrelation for the OLS model (the value of Moran’s I ranging from -0.22 to 0.25 and from -0.09 to 0.175 for M21 and M63, respectively). The residuals of GWR had a lower range of Moran’s I, i.e., between -0.05 and 0.02, indicating a lack of spatial autocorrelation.

## Discussion

### Model evaluation and spatial non-stationarity

A comparison of the inter-quartile range at the local level and of the standard error at the global level is useful in examining the non-stationarity. A parameter is said to be non-stationary when the inter-quartile range of the local model is greater than twice the standard error (SE) of the global model [25]. We explored the effect of spatial scale on the relationship between species richness and climate by varying the bandwidth used in the GWR analysis. At a coarser bandwidth, the spatial patterns of the local estimates become generalized and the estimated parameters mimic the estimates of global models more closely. This is supposed to be due to a decrease in the number of degrees of freedom in local model calibration leading to unstable regression results. The size of the spatial unit or kernel in relation to the true scale of spatial variation determines the homogeneity at a local scale. A lower bandwidth revealed more geographic details because of localisation [25]. Our results support the notion that global models (OLS) may ignore the assumption of heterogeneity in large-scale assessments as they may fail to depict the actual effects of variables [41, 42]. GWR analysis accommodates spatial heterogeneity, allowing the model parameters to vary in space, thus representing a stronger relationship between climate and species richness that is closer to reality.

### Spatial climate heterogeneity-linked plant richness

Climate is known to change along geographic and elevational gradients, and the plant richness found at different gradients is differentially adapted to these varying conditions [43]. Our results showed that the spatial heterogeneity of climate variables is important for explaining the biogeographic distribution of species richness, revealing the dominance of water, energy, or both variables. Variation partitioning revealed a strong influence of water (Pmin) on species richness in the Deccan peninsula and Western Ghats zones, in the lower latitudes (Figs 3iii & 4biii). However, an influence of both variables, i.e. water and energy, was prominent in the arid and semi-arid zones. The North-eastern zone, the Deccan peninsula and the Western Ghats zone have a typical tropical climate with high temperatures (ample energy) and rainfall. A gradual decrease in species richness from the south to the north in the Western Ghats and in the Eastern Ghats of the Deccan peninsula shows a linear relationship with Pmin. Southern India has tropical vegetation in a narrower climatic range. The requirement of precipitation for germination varies in tropics for varied storey species i.e. upper canopy, middle and understorey. However, the minimum requirement of precipitation necessary for germination is sufficiently available in the tropics. In contrast, the very low to moderate species richness of the arid and semi-arid zones could be explained by the limited availability of soil moisture for germination [44]. These zones have the high annual temperature ranges and low, erratic rainfall that characterise the harsh climate of these zones (S2 and S3 Figs). The availability of water strongly limits plant growth by directly influencing the physiological tolerance of plants [44].

The shift in the climate variables and their power to explain the species richness of the bio–geographic zones suggest that the climate–diversity relationships of plants species vary spatially. In particular, the dominant influence of Tmin and Pmin could be linked closely to the CTH. The CTH hypothesis of environmental filtering suggests that environments with suitable temperatures and water availability have greater species richness because more species can tolerate benign climates [45]. However, regional pools of species can persist in a local community if they can overcome site-specific environmental challenges, i.e. drought, cold or any kind of stress, whereas this filtering removes the intolerant species in more xeric conditions [46, 47]. The influence of precipitation variables in Deccan peninsula and the Western Ghats zone is ecologically meaningful since abundant availability of water and optimum temperatures enhance photosynthesis, leading to more intense biological activities and thus to high species richness. The diversity in a region where water is freely available could also be enhanced due to the competitive advantage of shade-tolerant species that enhance the under-storey species pool [48, 49]. In contrast, the available moisture at and near the soil surface is critical for desert plants. The availability of moisture depends on the balance between the input from precipitation and losses from evapo-transpiration. In addition, frequent and large fluctuations in temperature could cause the plants to adjust their physiological status within a very short time to cope with extremes, i.e. from very high to very low temperatures. These cumulative effects of climate extremes could lead to the lower species richness in the desert and the semi-arid zone as fewer species are physiologically adapted to persist in this environment. The smaller ranges of Pmin (0.41 mm to 94 mm) in these zones, along with the larger ranges of Tmax (22°C to 35.7°C) and MAT (12.8°C to 27.2°C) within 100 years (S3 and S4 Figs) could have amplified the influence of the climatic harshness, resulting in lower soil moisture levels and lower species richness. Goldie et al. [50] reported that woody plants in the arid Australian outback are evolving more slowly than are the species growing in moist regions at similar latitudes due to water limitation. Wohlgemuth et al. [51] observed that precipitation below a minimum annual threshold decreased the forest species richness in Switzerland. Brodribb et al. [52] observed minimum rainfall to be most strongly related to the photosynthetic activities of conifers, suggesting that the distributions of these species are constrained by the drought tolerance of their photosynthetic apparatus. In general, rainfall has been observed to be the major determinant of the large-scale distribution patterns of tropical plant species [53–55]. In general, higher densities of tree species in tropics could be expected due to the higher rainfall [56]. This coexistence of various species and the higher species density may be facilitated by the absence of competition for moisture.

In the North-east zone, the stronger correlation of precipitation with a moderate to a high species richness could be attributed to a wide range of climate gradients resulting from the complex topography. This variation results in great habitat heterogeneity, producing ‘ecoclines’ due to variations in precipitation and temperature. Lomolino [57] pointed out that variations in climate components, i.e. precipitation and temperature, along the elevation gradients might create variations in species richness. The cumulative impact of variations in non-climatic variables such as insulation, aspect and topography results in greater vegetation gradients (coenoclines) and thus enhances the local micro-climate [58]. Therefore, we may expect a significant influence of other climate and environmental variables, i.e. elevation and heterogeneity, on species in this zone. Our results are in line with those of Li et al. [59], who found water availability to be a major factor controlling species richness in dry lands of China. Similarly, Robertson et al. [60] have shown the significant contributions of summer and winter precipitation to the patterns of species richness in grasslands of the Chihuahuan desert, where they observed increased community-level species richness with increasing winter precipitation.

## Conclusions

The key finding from our study is that climate heterogeneity underlies the broad scale species richness distribution in India. We suggest that the spatial distribution of climatic water availability strongly influences the distribution of species in India. Species richness predictors are thought to vary systematically with the spatial scale at which climate–richness relationships are quantified. In the present study, the GWR bandwidth defined this scale. Although we found climate variables to have a decisive influence in defining species richness patterns in India, there are various other environmental and non-environmental (edaphic, topographic and anthropogenic) variables that need to be integrated in the models to understand climate–species richness relationships better at a finer grid level. The data were analysed at the 1° grid level, and only a few grid cells were available in the Himalaya (18) and in the Western Ghats zone (17). Therefore, elevational variations were not considered in the present study. The Gangetic plains, the Himalaya and the Trans-Himalaya need critical analysis. The moderate to high species richness in these zones were overlapped with the presence of protected areas, which is highlighting the potential contributions of these zones to the species pool, but constrained by forest loss. India has some of the most diverse bio-climatic zones of the world; nonetheless, the large human population and extensive agricultural activities exert continuous pressure on the forests, leading to decreasing species richness. The warming of bio-climatic regions, especially the potential effects of changes in precipitation, needs to be investigated closely to understand climate–species richness relationships so that mitigation measures may be developed in the face of climate change.

## Supporting information

S1 Fig (a) Plant species distribution in India represented at 1^0^ x 1^0^ grids (adapted from Tripathi et al., 2017); (b) a representation of modified nested quadrate showing tree (20m), shrub (5m) and herb (1m) sample plots. (TIF)

S2 Fig Spatial correlograms for (i) precipitation and (ii) temperature variables. (TIF)

S3 Fig Temperature (in °C) averaged over100 years showing (i) minimum, (ii) average and (iii) maximum temperature. * **Minimum temperature**: mean temperature of the coldest month (Tmin) **Average temperature**: mean annual temperature (MAT), **Maximum temperature**: Mean temperature of the warmest month (Tmax).
(TIF)

S4 Fig Precipitation data (in mm) averaged over100 years showing (i) minimum, (ii) annual and (iii) maximum precipitation. * **Minimum precipitation**: precipitation of the driest month (Pmin) **Annual precipitation**: mean annual precipitation (MAP), **Maximum precipitation**: mean and mean precipitation of the wettest month (Pmax).
(TIF)

S5 Fig (a) Temperature and (b) precipitation anomaly from 1901 to 2000. (TIF)

S1 Table Statistical analysis showing AICc and R^2^ values for precipitation and temperature (with 6-variables and their combination) with species richness as derived from OLS and GWR (at different % neighbour) models. (DOCX)

S2 Table Zone wise plant species richness list and plots for India at 1° grid level. (DOCX)

S1 Appendix (i) Methodology for anomaly derivation (ii) climate variables. (DOCX)
