# Supplementary figures and images for: Spatial heterogeneity of climate explains plant richness distribution at the regional scale in India
Source: PLoS One. 2019 Jun 20;14(6):e0218322. doi: 10.1371/journal.pone.0218322 (PMC6586307; doi:10.1371/journal.pone.0218322)

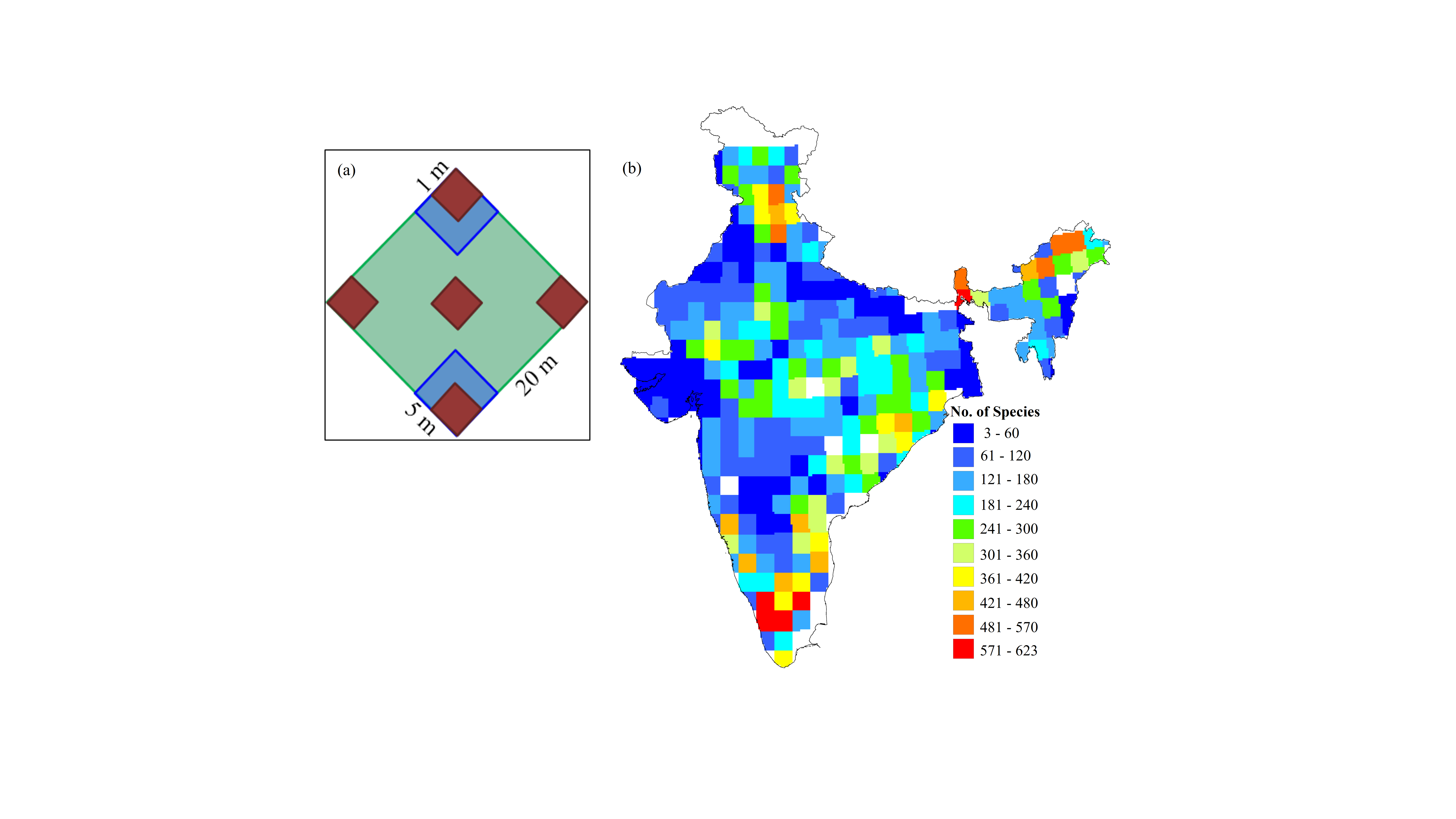

Supplement: S1 Fig — (TIF) [file pone.0218322.s001.tif]

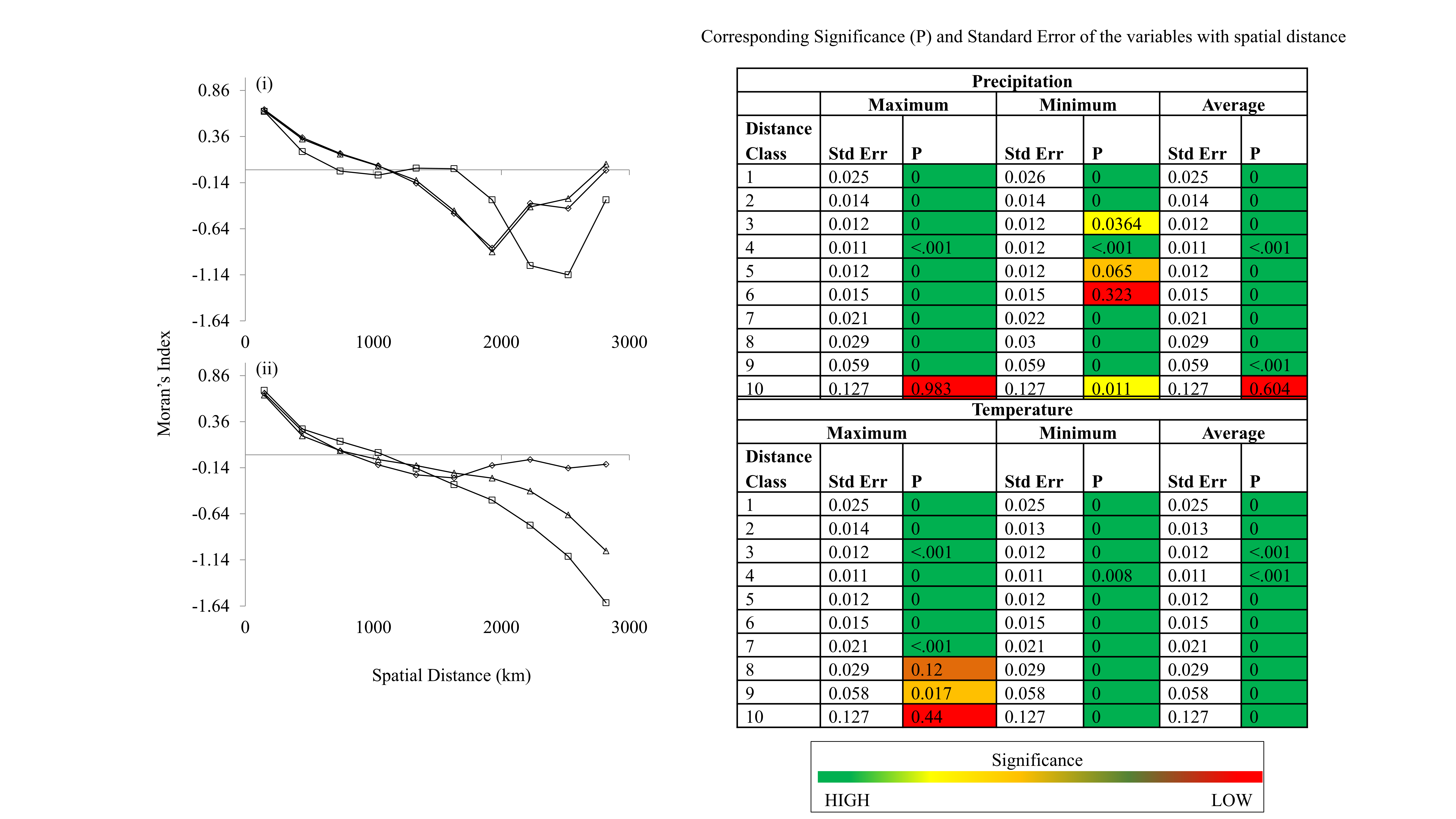

Supplement: S2 Fig — (TIF) [file pone.0218322.s002.tif]

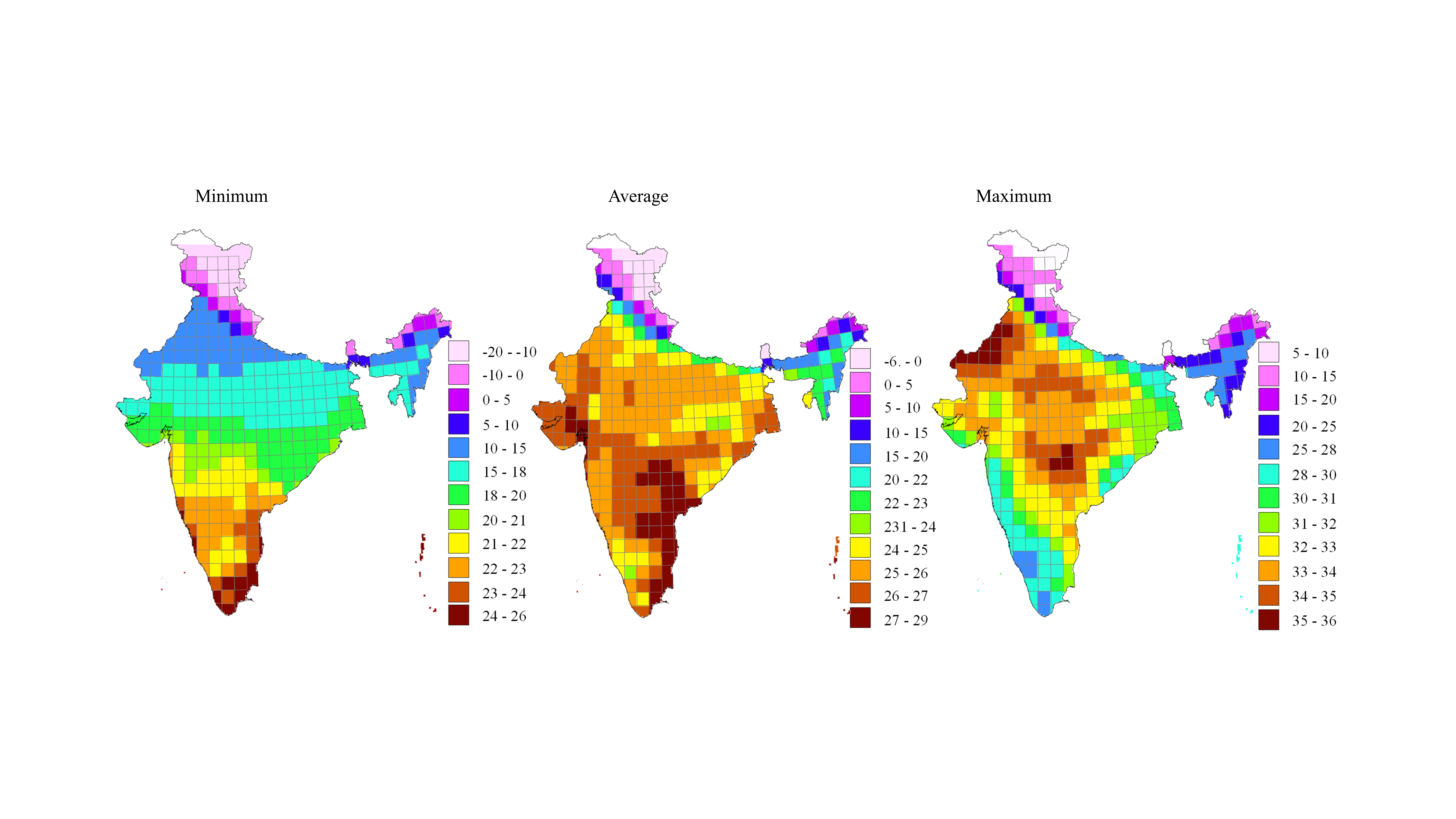

Supplement: S3 Fig — * Minimum temperature: mean temperature of the coldest month (Tmin) Average temperature: mean annual temperature (MAT), Maximum temperature: Mean temperature of the warmest month (Tmax). (TIF) [file pone.0218322.s003.tif]

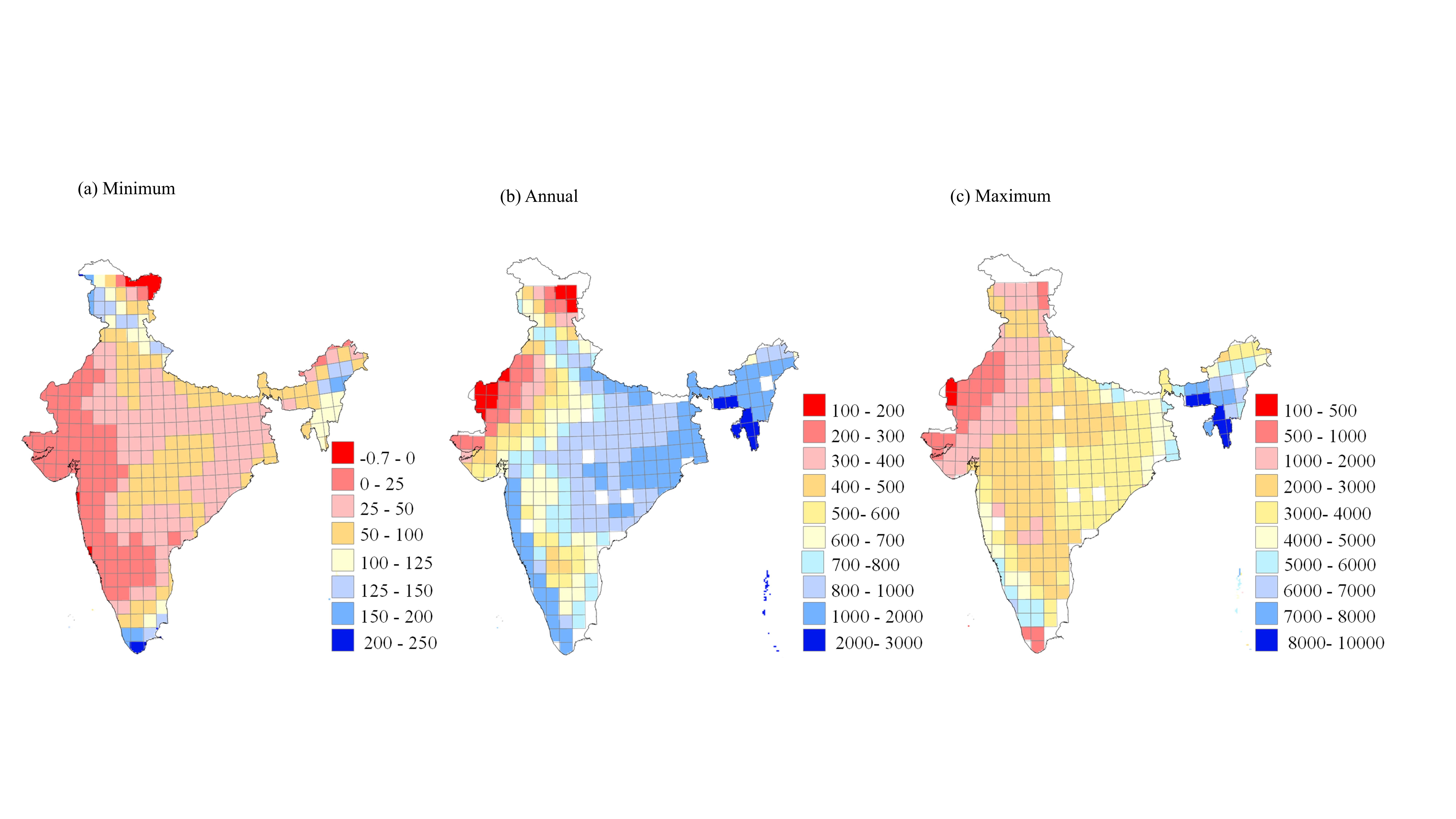

Supplement: S4 Fig — * Minimum precipitation: precipitation of the driest month (Pmin) Annual precipitation: mean annual precipitation (MAP), Maximum precipitation: mean and mean precipitation of the wettest month (Pmax). (TIF) [file pone.0218322.s004.tif]

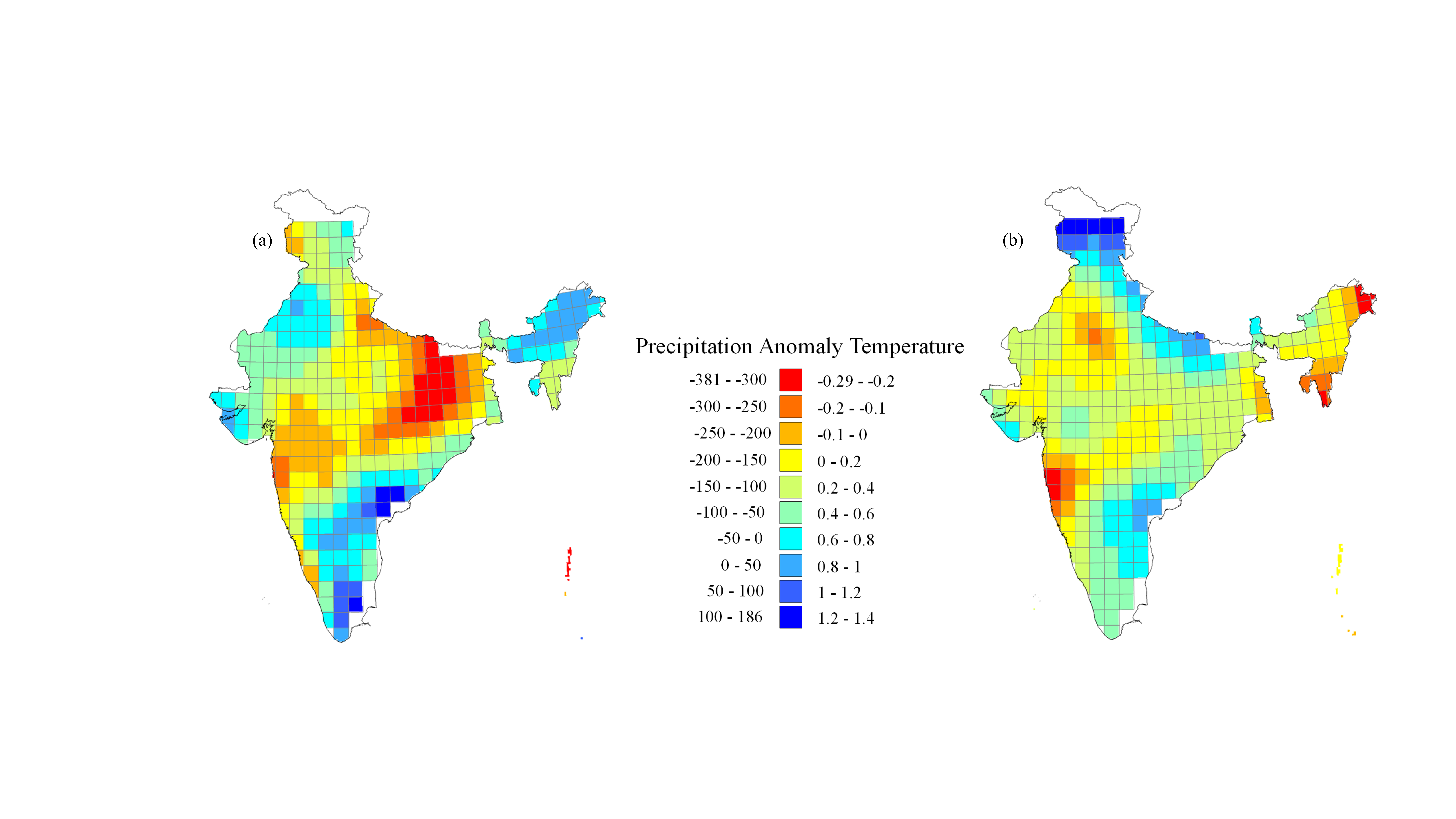

Supplement: S5 Fig — (TIF) [file pone.0218322.s005.tif]
