# Supplementary material for: Spatial heterogeneity of climate explains plant richness distribution at the regional scale in India
Source: PLoS One. 2019 Jun 20;14(6):e0218322. doi: 10.1371/journal.pone.0218322 (PMC6586307; doi:10.1371/journal.pone.0218322)
Supplement: S1 Table — (DOCX) [file pone.0218322.s006.docx]

S1 Table Statistical analysis showing AICc and R^2^ values for precipitation and temperature (with 6-variables and their combination) with species richness as derived from OLS and GWR (at different % neighbour) models

|  |  | **OLS** | | **GWR (neighbour %)** | | | | | | | |
| --- | --- | --- | --- | --- | --- | --- | --- | --- | --- | --- | --- |
|  |  |  |  | **5%** |  | **10%** |  | **15%** |  | **20%** |  |
| Variable combination | Combination/Model Number | AICc | R^2^ | AICc | R^2^ | AICc | R^2^ | AICc | R^2^ | AICc | R^2^ |
| T_avg_ | Model 1 | 2744.3 | 0.04 | 2681.0 | 0.78 | 2653.0 | 0.63 | 2666.0 | 0.53 | 2671.6 | 0.47 |
| P_avg_ | Model 2 | 2754.0 | 0.00 | 2671.5 | 0.77 | 2662.2 | 0.61 | 2676.6 | 0.50 | 2684.6 | 0.45 |
| T_max_ | Model 3 | 2742.6 | **0.05** | 2676.2 | 0.79 | 2643.5 | 0.64 | 2656.1 | 0.54 | 2663.0 | 0.49 |
| T_min_ | Model 4 | 2751.3 | 0.01 | 2655.3 | 0.80 | **2635.9** | **0.64** | 2651.5 | 0.54 | 2660.2 | 0.48 |
| P_max_ | Model 5 | 2752.7 | 0.01 | 2657.1 | 0.80 | 2652.8 | 0.62 | 2669.6 | 0.51 | 2678.6 | 0.45 |
| P_min_ | Model 6 | 2747.2 | 0.03 | 2653.4 | 0.81 | **2608.0** | **0.69** | 2619.4 | 0.61 | 2636.8 | 0.54 |
| Tavg+Tmax | Model 7 | 2744.5 | 0.05 | 2746.5 | 0.84 | 2648.1 | 0.69 | 2652.9 | 0.59 | 2655.1 | 0.53 |
| Tavg+Tmin | Model 8 | 2730.2 | **0.11** | 2744.5 | 0.83 | 2656.6 | 0.69 | 2666.0 | 0.57 | 2670.6 | 0.50 |
| Tmax+Tmin | Model 9 | 2740.1 | 0.07 | 2739.4 | 0.84 | **2647.5** | **0.69** | 2655.2 | 0.58 | 2657.2 | 0.53 |
| Pavg+Pmax | Model 10 | 2749.7 | 0.03 | 2755.9 | 0.83 | 2656.3 | 0.68 | 2660.9 | 0.59 | 2668.2 | 0.52 |
| Pavg+Pmin | Model 11 | 2749.2 | 0.03 | 2733.3 | 0.84 | 2625.2 | 0.71 | 2623.6 | 0.64 | 2637.1 | 0.57 |
| Pmax+Pmin | Model 12 | 2749.1 | 0.03 | 2724.5 | 0.85 | 2625.1 | 0.71 | 2621.5 | 0.64 | 2632.5 | 0.58 |
| Tavg+Pavg | Model 13 | 2745.9 | 0.05 | 2769.1 | 0.82 | 2664.9 | 0.67 | 2674.3 | 0.56 | 2676.7 | 0.50 |
| Tavg+Pmax | Model 14 | 2744.5 | 0.05 | 2778.8 | 0.82 | 2668.1 | 0.67 | 2672.3 | 0.56 | 2672.8 | 0.50 |
| Tavg+Pmin | Model 15 | 2745.3 | 0.05 | 2746.0 | 0.84 | 2631.8 | 0.71 | 2631.4 | 0.63 | 2635.9 | 0.58 |
| Tmax+Pavg | Model 16 | 2744.7 | 0.05 | 2765.1 | 0.82 | 2659.0 | 0.68 | 2668.1 | 0.57 | 2673.2 | 0.51 |
| Tmax+Pmax | Model 17 | 2744.2 | 0.05 | 2776.1 | 0.82 | 2666.8 | 0.67 | 2669.7 | 0.56 | 2672.1 | 0.50 |
| Tmax+Pmin | Model 18 | 2743.7 | 0.06 | 2733.3 | 0.84 | 2625.5 | 0.72 | 2624.2 | 0.64 | 2631.7 | 0.59 |
| Tmin+Pavg | Model 19 | 2752.6 | **0.02** | 2734.2 | 0.83 | 2655.1 | 0.67 | 2663.0 | 0.56 | 2666.5 | 0.50 |
| Tmin+Pmax | Model 20 | 2750.7 | 0.03 | 2735.7 | 0.84 | 2656.6 | 0.66 | 2660.6 | 0.56 | 2663.1 | 0.50 |
| Tmin+Pmin | Model 21 | 2749.21 | 0.03 | 2716.14 | 0.85 | **2619.6** | **0.72** | 2619.66 | 0.64 | 2626.70 | 0.58 |
| Tavg+Tmax +Tmin | Model 22 | 2728.9 | **0.13** | 2856.4 | 0.89 | 2666.3 | 0.73 | 2665.5 | 0.61 | 2662.7 | 0.55 |
| Tavg+Tmax+ Pmin | Model 23 | 2745.4 | 0.06 | 2891.3 | 0.88 | 2650.3 | 0.75 | 2630.3 | 0.68 | **2623.8** | **0.63** |
| Tmax+Tmin+Pavg | Model 24 | 2740.2 | 0.08 | 2863.5 | 0.89 | 2677.3 | 0.72 | 2674.3 | 0.60 | 2672.8 | 0.53 |
| Tavg+Tmin+Pavg | Model 25 | 2730.0 | 0.12 | 2909.6 | 0.87 | 2689.9 | 0.70 | 2683.3 | 0.59 | 2681.5 | 0.52 |
| Tavg+Tmin+Pmax | Model 26 | 2731.2 | 0.12 | 2878.8 | 0.89 | 2646.2 | 0.75 | 2631.2 | 0.67 | 2627.9 | 0.62 |
| Tavg+Tmax+Pmax | Model 27 | 2746.2 | 0.05 | 2885.5 | 0.90 | 2672.2 | 0.73 | 2670.1 | 0.61 | 2665.8 | 0.55 |
| Tmax+Tmin+Pmax | Model 28 | 2741.4 | 0.08 | 2892.8 | 0.89 | 2682.2 | 0.72 | 2677.7 | 0.59 | 2672.5 | 0.53 |
| Tavg+Pavg+Pmax | Model 29 | 2742.2 | 0.07 | 2980.0 | 0.86 | 2690.2 | 0.71 | 2683.0 | 0.60 | 2680.8 | 0.53 |
| Tavg+Pmax+Pmin | Model 30 | 2746.3 | 0.05 | 2894.4 | 0.88 | 2655.7 | 0.75 | 2639.0 | 0.67 | 2639.7 | 0.61 |
| Tmax+Pavg+Pmax | Model 31 | 2742.4 | 0.07 | 2964.0 | 0.86 | 2693.3 | 0.72 | 2682.0 | 0.61 | 2679.4 | 0.54 |
| Tmax+Pmax+Pmin | Model 32 | 2745.6 | 0.06 | 2886.5 | 0.88 | 2654.0 | 0.75 | 2636.4 | 0.67 | 2637.3 | 0.61 |
| Tmin+Pavg+Pmax | Model 33 | 2747.8 | 0.05 | 2932.6 | 0.87 | 2681.1 | 0.71 | 2672.5 | 0.60 | 2670.4 | 0.54 |
| Tmin+Pmax+Pmin | Model 34 | 2750.8 | 0.03 | 2862.9 | 0.90 | 2647.5 | 0.75 | 2630.4 | 0.67 | 2632.9 | 0.61 |
| Pavg+Pmax+Pmin | Model 35 | 2745.4 | 0.06 | 2918.3 | 0.87 | 2644.8 | 0.75 | **2626.4** | **0.68** | **2634.2** | **0.62** |
| Tavg+Tmax+Pavg | Model 36 | 2746.6 | 0.05 | 2903.1 | 0.88 | 2670.9 | 0.72 | 2669.1 | 0.61 | 2666.3 | 0.55 |
| Tavg+Pavg+Pmin | Model 37 | 2747.3 | 0.05 | 2903.1 | 0.88 | 2651.8 | 0.74 | 2639.7 | 0.66 | 2640.4 | 0.61 |
| Tmax+Pavg+Pmin | Model 38 | 2745.8 | 0.06 | 2884.8 | 0.87 | 2651.5 | 0.74 | 2635.4 | 0.67 | 2637.5 | 0.61 |
| Tmin+Pavg+Pmin | Model 39 | 2751.3 | **0.03** | 2877.2 | 0.90 | 2648.3 | 0.74 | 2630.6 | 0.66 | 2634.6 | 0.60 |
| Tavg+Tmin+ Pmin | Model 40 | **2730.9** | 0.12 | 2878.8 | 0.89 | 2646.2 | 0.75 | 2631.2 | 0.67 | 2627.9 | 0.62 |
| Tmax+Tmin+Pmin | Model 41 | 2740.0 | 0.08 | 2863.1 | 0.89 | 2640.2 | 0.75 | **2622.4** | **0.68** | **2618.5** | **0.64** |
| Tavg+Tmax+Tmin+Pmax | Model 42 | 2731.0 | 0.13 | 3169.0 | 0.94 | 2704.4 | 0.76 | 2686.7 | 0.63 | 2671.7 | 0.58 |
| Tavg+Tmax+Pavg+Pmax | Model 43 | 2744.3 | 0.07 | 3274.0 | 0.92 | 2708.8 | 0.76 | 2682.4 | 0.65 | 2672.6 | 0.59 |
| Tavg+Tmax+Pmin+Pmax | Model 44 | 2747.5 | 0.06 | 3122.0 | 0.94 | 2677.3 | 0.79 | 2643.1 | 0.71 | **2631.4** | **0.66** |
| Tavg+Tmin+Pavg+Pmax | Model 45 | 2730.9 | 0.13 | 3285.5 | 0.90 | 2721.3 | 0.74 | 2695.0 | 0.63 | 2685.6 | 0.56 |
| Tavg+Tmin+Pmin+Pmax | Model 46 | 2729.6 | 0.13 | 3149.7 | 0.94 | 2677.2 | 0.78 | 2644.9 | 0.70 | 2636.7 | 0.65 |
| Tavg+Pavg+Pmin+Pmax | Model 47 | 2743.7 | 0.07 | 3240.1 | 0.91 | 2686.1 | 0.78 | 2652.0 | 0.70 | 2649.2 | 0.64 |
| Tmax+Pavg+Pmin+Pmax | Model 48 | 2743.4 | 0.08 | 3242.8 | 0.91 | 2687.6 | 0.78 | 2649.2 | 0.70 | 2644.1 | 0.64 |
| Tmin+Pavg+Pmin+Pmax | Model 49 | 2747.3 | 0.06 | 3227.7 | 0.92 | 2687.2 | 0.77 | 2645.7 | 0.70 | 2642.6 | 0.64 |
| Tavg+Tmax+Tmin+Pavg | Model 50 | 2730.4 | 0.13 | 3143.6 | 0.93 | 2705.5 | 0.75 | 2682.2 | 0.63 | 2668.2 | 0.58 |
| Tavg+Tmax+Tmin+Pmin | Model 51 | 2729.87 | 0.1 | 3190.51 | 0.9 | 2670.69 | 0.8 | 2641.66 | 0.7 | **2629.2** | **0.66** |
| Tavg+Tmax+Pavg+ Pmin | Model 52 | 2746.8 | **0.06** | 3175.2 | 0.93 | 2674.8 | 0.78 | 2642.1 | 0.70 | **2631.4** | **0.66** |
| Tavg+Tmin+Pavg+ Pmin | Model 53 | 2727.5 | **0.14** | 3145.9 | 0.94 | 2678.6 | 0.77 | 2644.9 | 0.70 | 2638.3 | 0.64 |
| Tmax+Tmin+Pavg+Pmin | Model 54 | 2735.4 | 0.11 | 3131.0 | 0.93 | 2677.8 | 0.78 | 2642.1 | 0.70 | 2634.0 | 0.65 |
| Tmax+Tmin+Pavg+Pmax | Model 55 | 2735.4 | 0.11 | 3216.1 | 0.93 | 2716.7 | 0.76 | 2689.1 | 0.64 | 2679.2 | 0.57 |
| Tmax+Tmin+Pmax+Pmin | Model 56 | 2737.8 | 0.10 | 3147.2 | 0.94 | 2678.2 | 0.79 | 2644.8 | 0.70 | 2635.3 | 0.65 |
| Tavg+Pavg+Tmin+Tmax+Pmin | Model 57 | 2729.1 | 0.14 | 3678.5 | 0.97 | 2703.2 | 0.82 | 2657.9 | 0.73 | 2639.9 | 0.68 |
| Tavg+Tmax+ Tmin+Pavg+Pmax | Model 58 | 2730.0 | 0.14 | 3846.7 | 0.96 | 2750.0 | 0.79 | 2696.5 | 0.68 | 2673.6 | 0.62 |
| Tavg+Tmax+Tmin+Pmin+Pmax | Model 59 | 2730.9 | 0.14 | 3665.7 | 0.97 | 2707.2 | 0.83 | 2660.4 | 0.73 | 2642.5 | 0.68 |
| Tavg+Tmax+Pavg+Pmin+Pmax | Model 60 | 2745.5 | **0.08** | 3794.2 | 0.96 | 2714.9 | 0.82 | 2654.6 | 0.74 | **2637.2** | **0.69** |
| Tavg+Tmin+Pavg+Pmin+Pmax | Model 61 | 2728.9 | **0.14** | 3862.1 | 0.96 | 2717.8 | 0.81 | 2660.0 | 0.73 | 2645.4 | 0.67 |
| Tmax+Tmin+Pavg+Pmin+Pmax | Model 62 | 2737.1 | 0.11 | 3814.1 | 0.96 | 2723.4 | 0.81 | 2656.7 | 0.74 | 2639.5 | 0.68 |
| Tavg+Tmax+Tmin+Pavg+Pmin+Pmax | Model 63 | 2729.9 | 0.15 | 4917.3 | 0.97 | 2760.5 | 0.86 | 2677.6 | 0.76 | **2649.2** | **0.70** |
