# Supplementary material for: Spatial heterogeneity of climate explains plant richness distribution at the regional scale in India
Source: PLoS One. 2019 Jun 20;14(6):e0218322. doi: 10.1371/journal.pone.0218322 (PMC6586307; doi:10.1371/journal.pone.0218322)
Supplement: S2 Table — (DOCX) [file pone.0218322.s007.docx]

S2 Table Zone wise plant species richness list and plots for India at 1˚ grid level

| **Deccan Peninsula** | | | | | | | | | | | | | | |
| --- | --- | --- | --- | --- | --- | --- | --- | --- | --- | --- | --- | --- | --- | --- |
| **S. No.** | | | **Species** | | | **Plot** | | | **Vegetation type** | | | **Vegetation %** | | |
| 1 | | | 133 | | | 38 | | | 4 | | | 6.78 | | |
| 2 | | | 94 | | | 18 | | | 4 | | | 7.58 | | |
| 3 | | | 141 | | | 59 | | | 4 | | | 24.29 | | |
| 4 | | | 186 | | | 134 | | | 12 | | | 41.44 | | |
| 5 | | | 123 | | | 67 | | | 9 | | | 33.11 | | |
| 6 | | | 231 | | | 67 | | | 5 | | | 37.88 | | |
| 7 | | | 303 | | | 161 | | | 9 | | | 47.07 | | |
| 8 | | | 237 | | | 114 | | | 14 | | | 40.99 | | |
| 9 | | | 161 | | | 109 | | | 6 | | | 28.02 | | |
| 10 | | | 291 | | | 89 | | | 6 | | | 33.25 | | |
| 11 | | | 141 | | | 55 | | | 5 | | | 43.3 | | |
| 12 | | | 254 | | | 91 | | | 9 | | | 36.14 | | |
| 13 | | | 229 | | | 40 | | | 5 | | | 71.21 | | |
| 14 | | | 217 | | | 44 | | | 6 | | | 39.17 | | |
| 15 | | | 355 | | | 82 | | | 5 | | | 50.71 | | |
| 16 | | | 106 | | | 105 | | | 6 | | | 44.71 | | |
| 17 | | | 221 | | | 58 | | | 7 | | | 50.43 | | |
| 18 | | | 295 | | | 105 | | | 9 | | | 44.42 | | |
| 19 | | | 245 | | | 67 | | | 6 | | | 36.81 | | |
| 20 | | | 268 | | | 49 | | | 9 | | | 30.11 | | |
| 21 | | | 255 | | | 50 | | | 10 | | | 35.19 | | |
| 22 | | | 189 | | | 110 | | | 7 | | | 38.7 | | |
| 23 | | | 189 | | | 126 | | | 11 | | | 35.3 | | |
| 24 | | | 258 | | | 70 | | | 7 | | | 30.04 | | |
| 25 | | | 283 | | | 236 | | | 8 | | | 59.14 | | |
| 26 | | | 198 | | | 48 | | | 6 | | | 59.01 | | |
| 27 | | | 396 | | | 208 | | | 10 | | | 44.64 | | |
| 28 | | | 124 | | | 36 | | | 8 | | | 13.5 | | |
| 29 | | | 116 | | | 36 | | | 6 | | | 8.75 | | |
| 30 | | | 131 | | | 76 | | | 4 | | | 14.85 | | |
| 31 | | | 144 | | | 196 | | | 7 | | | 34.74 | | |
| 32 | | | 140 | | | 78 | | | 6 | | | 55.92 | | |
| 33 | | | 251 | | | 97 | | | 7 | | | 48.88 | | |
| 34 | | | 462 | | | 199 | | | 9 | | | 64.2 | | |
| 35 | | | 272 | | | 105 | | | 10 | | | 33.28 | | |
| 36 | | | 128 | | | 23 | | | 5 | | | 5.7 | | |
| 37 | | | 114 | | | 25 | | | 6 | | | 3.15 | | |
| 38 | | | 73 | | | 47 | | | 7 | | | 39.07 | | |
| 39 | | | 110 | | | 50 | | | 7 | | | 43.49 | | |
| 40 | | | 203 | | | 49 | | | 8 | | | 55.37 | | |
| 41 | | | 354 | | | 192 | | | 9 | | | 70.64 | | |
| 42 | | | 414 | | | 208 | | | 11 | | | 55.22 | | |
| 43 | | | 71 | | | 9 | | | 2 | | | 11.74 | | |
| 44 | | | 109 | | | 23 | | | 6 | | | 9 | | |
| 45 | | | 91 | | | 12 | | | 5 | | | 5.43 | | |
| 46 | | | 308 | | | 104 | | | 9 | | | 74.25 | | |
| 47 | | | 211 | | | 38 | | | 8 | | | 12.2 | | |
| 48 | | | 208 | | | 125 | | | 11 | | | 57.5 | | |
| 49 | | | 254 | | | 75 | | | 8 | | | 37.12 | | |
| 50 | | | 46 | | | 8 | | | 1 | | | 7.69 | | |
| 51 | | | 266 | | | 87 | | | 5 | | | 25.49 | | |
| 52 | | | 476 | | | 153 | | | 9 | | | 35.89 | | |
| 53 | | | 100 | | | 20 | | | 3 | | | 11.9 | | |
| 54 | | | 345 | | | 77 | | | 5 | | | 26.95 | | |
| 55 | | | 402 | | | 87 | | | 6 | | | 40.24 | | |
| 56 | | | 73 | | | 11 | | | 3 | | | 24.11 | | |
| 57 | | | 449 | | | 105 | | | 11 | | | 42.13 | | |
| 58 | | | 460 | | | 132 | | | 13 | | | 42.64 | | |
| 59 | | | 389 | | | 66 | | | 10 | | | 32.1 | | |
| 60 | | | **609** | | | 242 | | | 14 | | | 31.48 | | |
| **S. No.** | | **Species** | | | **Plot** | | | **Vegetation type** | | | **Vegetation %** | | |  |
| 61 | | 99 | | | 27 | | | 9 | | | 10.14 | | |  |
| 62 | | 143 | | | 50 | | | 10 | | | 44.78 | | |  |
| 63 | | 178 | | | 73 | | | 9 | | | 30.18 | | |  |
| 64 | | 156 | | | 68 | | | 7 | | | 26.35 | | |  |
| 65 | | 350 | | | 137 | | | 8 | | | 63.23 | | |  |
| 66 | | 233 | | | 49 | | | 7 | | | 57.87 | | |  |
| 67 | | 256 | | | 151 | | | 7 | | | 44.38 | | |  |
| 68 | | 147 | | | 90 | | | 6 | | | 44.58 | | |  |
| 69 | | 114 | | | 47 | | | 9 | | | 16.08 | | |  |
| 70 | | 335 | | | 87 | | | 9 | | | 46.28 | | |  |
| 71 | | 201 | | | 71 | | | 6 | | | 46.41 | | |  |
| 72 | | 163 | | | 60 | | | 7 | | | 57.55 | | |  |
| 73 | | 191 | | | 75 | | | 8 | | | 31.15 | | |  |
| 74 | | 158 | | | 170 | | | 11 | | | 42.87 | | |  |
| 75 | | 58 | | | 20 | | | 4 | | | 8.93 | | |  |
| 76 | | 145 | | | 88 | | | 5 | | | 21.49 | | |  |
| 77 | | 78 | | | 10 | | | 3 | | | 19.19 | | |  |
| 78 | | 102 | | | 28 | | | 7 | | | 11.07 | | |  |
| 79 | | 181 | | | 58 | | | 7 | | | 37.7 | | |  |
| 80 | | 394 | | | 146 | | | 7 | | | 38.02 | | |  |
| 81 | | 98 | | | 55 | | | 7 | | | 14.75 | | |  |
| 82 | | 40 | | | 15 | | | 1 | | | 21.54 | | |  |
| 83 | | 236 | | | 66 | | | 6 | | | 20.51 | | |  |
| 84 | | 276 | | | 55 | | | 9 | | | 48.05 | | |  |
| 85 | | 110 | | | 54 | | | 6 | | | 32.91 | | |  |
| 86 | | 48 | | | 8 | | | 2 | | | 4.57 | | |  |
| 87 | | 42 | | | 6 | | | 2 | | | 4.13 | | |  |
| 88 | | 168 | | | 105 | | | 8 | | | 37.54 | | |  |
| 89 | | 304 | | | 65 | | | 7 | | | 25.99 | | |  |
| 90 | | 99 | | | 25 | | | 5 | | | 15.61 | | |  |
| 91 | | 132 | | | 37 | | | 8 | | | 26.61 | | |  |
| 92 | | 383 | | | 96 | | | 8 | | | 37.32 | | |  |
| **Arid & semi-arid-zone** | | | | | | | | | | | | | | |
| **S. No.** | | | **Species** | | | **Plot** | | | **Vegetation type** | | | **Vegetation %** | | |
| 1 | | | 92 | | | 51 | | | 5 | | | 10.7 | | |
| 2 | | | 166 | | | 47 | | | 8 | | | 5.58 | | |
| 3 | | | 384 | | | 89 | | | 10 | | | 44.85 | | |
| 4 | | | **517** | | | 157 | | | 17 | | | 47.94 | | |
| 5 | | | 93 | | | 39 | | | 3 | | | 2.98 | | |
| 6 | | | 47 | | | 6 | | | 3 | | | 0.23 | | |
| 7 | | | 62 | | | 16 | | | 3 | | | 0.32 | | |
| 8 | | | 43 | | | 6 | | | 1 | | | 2.91 | | |
| 9 | | | 123 | | | 14 | | | 5 | | | 5.19 | | |
| 10 | | | 61 | | | 30 | | | 5 | | | 21.36 | | |
| 11 | | | 116 | | | 44 | | | 3 | | | 17.14 | | |
| 12 | | | 66 | | | 43 | | | 3 | | | 3.47 | | |
| 13 | | | 76 | | | 43 | | | 4 | | | 12.26 | | |
| 14 | | | 278 | | | 84 | | | 12 | | | 28 | | |
| 15 | | | 156 | | | 35 | | | 6 | | | 2.67 | | |
| 16 | | | 95 | | | 54 | | | 6 | | | 30.95 | | |
| 17 | | | 165 | | | 38 | | | 5 | | | 14.89 | | |
| 18 | | | 91 | | | 29 | | | 6 | | | 9.86 | | |
| 19 | | | 159 | | | 99 | | | 5 | | | 15.62 | | |
| 20 | | | 286 | | | 80 | | | 10 | | | 44.3 | | |
| 21 | | | 175 | | | 44 | | | 10 | | | 14.36 | | |
| 22 | | | 307 | | | 78 | | | 7 | | | 21.79 | | |
| 23 | | | 226 | | | 77 | | | 6 | | | 15.71 | | |
| 24 | | | 290 | | | 72 | | | 10 | | | 69.71 | | |
| 25 | | | 289 | | | 132 | | | 9 | | | 32.86 | | |
| 26 | | | 364 | | | 181 | | | 10 | | | 47.65 | | |
| 27 | | | 288 | | | 72 | | | 9 | | | 25.65 | | |
| 28 | | | 136 | | | 31 | | | 4 | | | 29.41 | | |
| 29 | | | 6 | | | 12 | | | 2 | | | 11.66 | | |
| 30 | | | 19 | | | 10 | | | 2 | | | 5 | | |
| 31 | | | 19 | | | 11 | | | 4 | | | 6.27 | | |
| 32 | | | 158 | | | 53 | | | 10 | | | 18.5 | | |
| 33 | | | 216 | | | 78 | | | 8 | | | 19.7 | | |
| 34 | | | 45 | | | 5 | | | 2 | | | 7.33 | | |
| 35 | | | 217 | | | 49 | | | 4 | | | 20.39 | | |
| 36 | | | **3** | | | 9 | | | 3 | | | 6.29 | | |
| 37 | | | 9 | | | 5 | | | 3 | | | 12.09 | | |
| 38 | | | 8 | | | 25 | | | 3 | | | 11.28 | | |
| 39 | | | 121 | | | 17 | | | 5 | | | 11.35 | | |
| 40 | | | 17 | | | 7 | | | 2 | | | 4.22 | | |
| 41 | | | 28 | | | 13 | | | 3 | | | 13.77 | | |
| 42 | | | 18 | | | 9 | | | 4 | | | 1.48 | | |
| 43 | | | 260 | | | 64 | | | 8 | | | 41.02 | | |
| 44 | | | 285 | | | 83 | | | 11 | | | 7.57 | | |
| 45 | | | 94 | | | 18 | | | 3 | | | 0.71 | | |
| 46 | | | 43 | | | 20 | | | 3 | | | 2.92 | | |
| 47 | | | 34 | | | 14 | | | 2 | | | 13.41 | | |
| 48 | | | 134 | | | 17 | | | 6 | | | 4.23 | | |
| 49 | | | 62 | | | 19 | | | 2 | | | 1.58 | | |
| 50 | | | 179 | | | 63 | | | 3 | | | 4.6 | | |
| 51 | | | 324 | | | 104 | | | 9 | | | 18.7 | | |
| 52 | | | 137 | | | 27 | | | 5 | | | 29.13 | | |
| 53 | | | 88 | | | 32 | | | 4 | | | 8.12 | | |
| 54 | | | 123 | | | 33 | | | 5 | | | 17.94 | | |
| 55 | | | 115 | | | 32 | | | 4 | | | 16.42 | | |
| 56 | | | 34 | | | 35 | | | 3 | | | 23.68 | | |
| 57 | | | 13 | | | 13 | | | 4 | | | 5.01 | | |
| 58 | | | 268 | | | 39 | | | 8 | | | 30.87 | | |
| 59 | | | 14 | | | 6 | | | 2 | | | 4.49 | | |

| **Gangetic plain** | | | | |
| --- | --- | --- | --- | --- |
| **S. No.** | **Species** | **Plot** | **Vegetation type** | **Vegetation %** |
| 1 | 38 | 5 | 2 | 2.1 |
| 2 | 64 | 24 | 8 | 5.45 |
| 3 | 66 | 27 | 7 | 4.82 |
| 4 | 43 | 9 | 5 | 4.35 |
| 5 | 50 | 20 | 3 | 6.05 |
| 6 | 95 | 25 | 4 | 5.15 |
| 7 | 86 | 33 | 4 | 8.94 |
| 8 | 69 | 17 | 5 | 1.58 |
| 9 | 128 | 39 | 5 | 1.79 |
| 10 | 28 | 6 | 1 | 1.88 |
| 11 | **623** | 167 | 12 | 18.41 |
| 12 | 315 | 74 | 9 | 21.39 |
| 13 | 77 | 17 | 2 | 2.29 |
| 14 | 130 | 36 | 6 | 8.82 |
| 15 | 82 | 26 | 2 | 10.2 |
| 16 | 90 | 68 | 6 | 18.94 |
| 17 | 51 | 37 | 4 | 32.3 |
| 18 | 106 | 87 | 8 | 18.55 |
| 19 | 28 | 8 | 3 | 1.48 |
| 20 | 167 | 58 | 5 | 6.93 |
| 21 | 77 | 13 | 2 | 0.3 |
| 22 | 40 | 8 | 2 | 6.3 |
| 23 | **24** | 10 | 2 | 5.8 |
| 24 | 26 | 6 | 2 | 14.25 |

| **Himalaya & trans-Himalaya zone** | | | | |
| --- | --- | --- | --- | --- |
| **S. No.** | **Species** | **Plot** | **Vegetation type** | **Vegetation %** |
| 1 | 136 | 70 | 12 | 59.7 |
| 2 | 238 | 93 | 7 | 51.4 |
| 3 | 234 | 50 | 5 | 19.8 |
| 4 | 177 | 38 | 2 | 33.5 |
| 5 | 264 | 131 | 5 | 32.1 |
| 6 | 155 | 35 | 3 | 18.8 |
| 7 | 453 | 100 | 8 | 79.4 |
| 8 | 362 | 74 | 4 | 41.7 |
| 9 | 240 | 144 | 13 | 62.7 |
| 10 | 142 | 37 | 8 | 27.9 |
| 11 | **531** | 210 | 16 | 41.2 |
| 12 | **73** | 24 | 6 | 8.97 |
| 13 | 267 | 66 | 3 | 33.31 |
| 14 | 277 | 114 | 12 | 61.39 |
| 15 | 145 | 45 | 7 | 71.55 |
| 16 | 488 | 188 | 6 | 26.04 |
| 17 | 134 | 35 | 9 | 67.91 |
| 18 | 131 | 28 | 6 | 6.99 |

| **North-east zone** | | | | |
| --- | --- | --- | --- | --- |
| **S. No.** | **Species** | **Plot** | **Vegetation type** | **Vegetation %** |
| 1 | **344** | 100 | 9 | 44.86 |
| 2 | 171 | 41 | 7 | 18.47 |
| 3 | 146 | 20 | 3 | 19.63 |
| 4 | 76 | 25 | 4 | 43.14 |
| 5 | 141 | 94 | 6 | 74.27 |
| 6 | 122 | 34 | 7 | 85.43 |
| 7 | 32 | 28 | 5 | 43.91 |
| 8 | 161 | 43 | 6 | 54.47 |
| 9 | 48 | 61 | 6 | 29.33 |
| 10 | 103 | 60 | 5 | 28.69 |
| 11 | 256 | 69 | 8 | 71.93 |
| 12 | 76 | 99 | 6 | 64.88 |
| 13 | 149 | 121 | 7 | 44.26 |
| 14 | 168 | 70 | 6 | 30.92 |
| 15 | **30** | 13 | 4 | 7.69 |

| **Western Ghats zone** | | | | |
| --- | --- | --- | --- | --- |
| **S. No.** | **Species** | **Plot** | **Vegetation type** | **Vegetation %** |
| 1 | **31** | 8 | 5 | 2.54 |
| 2 | 53 | 12 | 3 | 8.3 |
| 3 | 162 | 26 | 7 | 41.24 |
| 4 | 86 | 11 | 5 | 13.32 |
| 5 | 137 | 35 | 6 | 24 |
| 6 | 435 | 145 | 9 | 60.4 |
| 7 | 238 | 50 | 7 | 50.99 |
| 8 | 220 | 26 | 8 | 14.32 |
| 9 | 547 | 113 | 11 | 63.81 |
| 10 | **566** | 117 | 11 | 31.64 |
| 11 | 132 | 78 | 6 | 36.32 |
| 12 | 126 | 39 | 4 | 46.06 |
| 13 | 82 | 11 | 2 | 45.23 |
| 14 | 137 | 36 | 5 | 32.61 |
| 15 | 428 | 154 | 7 | 54.73 |
| 16 | 308 | 64 | 6 | 52.85 |
| 17 | 172 | 21 | 6 | 14.6 |
