# Supplementary material for: Spatial heterogeneity of climate explains plant richness distribution at the regional scale in India
Source: PLoS One. 2019 Jun 20;14(6):e0218322. doi: 10.1371/journal.pone.0218322 (PMC6586307; doi:10.1371/journal.pone.0218322)
Supplement: S1 Appendix — (DOCX) [file pone.0218322.s008.docx]

**S1 Appendix**

**(i) Methodology for Anomaly derivation**

To derive climate anomalies we have used mean based difference measures. For anomaly construction, we need to split data into two parts as (i) data with expected behaviour and (ii) data that shows variability from the expected. Here, the monthly mean values of temperature and precipitation were considered as the base showing the data with expected behaviour and these base values were subtracted from the individual raw data, which is a measure of deviation from expected. Therefore, for a given location i, its anomaly time series ƒ'i constructed from the raw time series *f*i by removing a base vector *f*i from it by following Kawale et al.(2011) and New et al. (1999) and given as:

ƒ'i = ƒi − (*f*i)

Where, ƒ'i represents the climate anomaly (deviation from the expected)

*f*i is the individual raw data for a particular location and (*f*i) is the base vector (represents the monthly mean values).

**(ii) Climate variables**

The range of Tmin varied between -20 to 26.8 ˚C wherein, lowest temperature (-20 to -10 ˚C) was observed in the Jammu & Kashmir and Sikkim states in the trans-Himalaya zone. These areas are covered with snow and mean temperatures are below 0 ˚C (S3 Fig. i & ii). The range of Tmax varies between 6 and 36 ˚C wherein maximum temperature (36 ˚C) was observed in the Bikaner and Jodhpur districts of Rajasthan in the Desert zone followed by Adilabad district of Andhra Pradesh and Chandrapur district of Maharashtra (35 ˚C) in Deccan Peninsula zone (Fig. S3, iii). The Pmin range varied between 0 and 86mm wherein, the lowest values were observed in the north-eastern Jammu & Kashmir in the trans-Himalaya zone and Amreli, Jamnagar and Rajkot districts of Gujarat state in arid zone (0 and 3mm). Climate data from these regions indicate arid conditions since these regions accommodate the cold desert in the trans-Himalaya and the great Rann of Kutch in the desert zone, one of the largest salt deserts of the world respectively. The western Himalaya zone is apparently only rarely affected by monsoonal precipitation of which, most would be snow in winter and spring. The range of Pmax varied between 16 and 1000mm for the country wherein, maximum values were observed in the east Khasi hills of Meghalaya and the Aizwal district of Mizoram in Northeast zone (Fig.S4, iii). Meghalaya is known for receiving maximum rainfall in the Mawsynram and Cherrapunji region receiving approx. 11,872 and 11,777mm of annual rainfall respectively. Climate anomaly data was derived for the study area following Kawale et al. (2011) that showed overall range between -0.2 and 1.4 ˚C for MAT and between -100 to 380mm for MAP respectively (Fig.S5; (See Appendix S3,i for anomaly derivation)). The maximum increase in MAT (0.8 to 1.4 ˚C) was observed in the Jammu Kashmir of the Himalaya zone and maximum decrease (0.2 ˚C) in the Maharashtra state of the Western Ghats zone respectively (Fig.S5,a). On the contrary, the maximum increase in precipitation (100-186mm) was observed in the grids of Andhra Pradesh, Odisha and Chhattisgarh states of the Deccan Peninsula zone. However, the maximum decrease (300 to 380mm) was observed in the grids of Bihar, Chhattisgarh, and Jharkhand states of Deccan Peninsula zone (Fig.S5, b).
